# Development of a Functional Glomerulus at the Organ Level on a Chip to Mimic Hypertensive Nephropathy

**DOI:** 10.1038/srep31771

**Published:** 2016-08-25

**Authors:** Mengying Zhou, Xulang Zhang, Xinyu Wen, Taihua Wu, Weidong Wang, Mingzhou Yang, Jing Wang, Ming Fang, Bingcheng Lin, Hongli Lin

**Affiliations:** 1 Department of Nephrology, The First Affiliated Hospital of Dalian Medical University, Key Laboratory of Kidney Disease of Liaoning Province, The Center for the Transformation Medicine of Kidney Disease of Liaoning Province, No. 222 Zhongshan Road, Dalian 116011, China; 2 Department of Biotechnology, Dalian Institute of Chemical Physics, Chinese Academy of Sciences, No. 457 Zhongshan Road, Dalian 116023, China; 3 Department of Urology, Dalian Friendship Hospital, No. 8 Sanba Square, Dalian, 116001, China

## Abstract

Glomerular hypertension is an important factor exacerbating glomerular diseases to end-stage renal diseases because, ultimately, it results in glomerular sclerosis (especially in hypertensive and diabetic nephropathy). The precise mechanism of glomerular sclerosis caused by glomerular hypertension is unclear, due partly to the absence of suitable *in vitro* or *in vivo* models capable of mimicking and regulating the complex mechanical forces and/or organ-level disease processes. We developed a “glomerulus-on-a-chip” (GC) microfluidic device. This device reconstitutes the glomerulus with organ-level glomerular functions to create a disease model-on-a chip that mimics hypertensive nephropathy in humans. It comprises two channels lined by closely opposed layers of glomerular endothelial cells and podocytes that experience fluid flow of physiological conditions to mimic the glomerular microenvironment *in vivo*. Our results revealed that glomerular mechanical forces have a crucial role in cellular cytoskeletal rearrangement as well as the damage to cells and their junctions that leads to increased glomerular leakage observed in hypertensive nephropathy. Results also showed that the GC could readily and flexibly meet the demands of a renal-disease model. The GC could provide drug screening and toxicology testing, and create potential new personalized and accurate therapeutic platforms for glomerular disease.

Hypertensive nephrology is a common kidney disease leading to end stage renal diseases. Glomerular hypertension is an important physio-pathologic characteristic that promotes kidney disease progression^1^,^2^. Systemic hypertension causes glomerular hypertension, which manifests as high perfusion pressure, high filtration pressure, and high transmembrane pressure in glomeruli^3^. The guidelines of the Kidney Disease Improving Global Outcomes (KDIGO) Foundation recommend angiotensin-converting enzyme inhibitors and angiotensin receptor blockers are used as first-line agents for kidney-disease patients with hypertension because of their unique role in reducing glomerular hypertension beyond decreasing systemic blood pressure^4^.

The precise mechanism by which glomerular hypertension induces glomerular injury and aids progression of nephrosclerosis is not clear. An important reason is that a suitable experimental model that can reconstitute hemodynamic factors in glomeruli quickly and reliably is not available. In existing animal models, precise regulation and monitoring of hemodynamic factors in glomeruli is difficult, expensive and time-consuming. In addition, *in vitro* models are used routinely to stimulate glomerular endothelial cells or podocytes separately by a single biomechanical factor (e.g., shear force, stretching force)^5^,^6^. These models have failed to provide integrated systems that replicate the complex and core physiological structure and functions of glomeruli, including the endothelial cells, basement membrane, and podocytes^7^, and place them in a dynamic, integrated, mechanical microenvironment.

A potential solution to this problem of the lack of glomerular models with a realistic microenvironment is the development of a human “organ-on-a-chip” (e.g., as has happened for the intestine, liver, heart, renal tubule, etc.). These are manufactured using microchip methods with microscale tissue-engineering technologies to simulate tissue- and organ-level physiology and mechanical microenvironments^8^,^9^,^10^,^11^,^12^,^13^,^14^,^15^,^16^. This intriguing approach has extensive application in the research and development of drugs. Recent production of a “lung-on-a-chip” and “liver-on-a-chip” could help to replace animal use for drug screening and toxicology testing^17^,^18^.

Some studies have produced functional renal tubular systems using microfluidic chips that are critical to improving early prediction of drug-induced kidney injury and drug screening. The majority of these systems consist of renal tubular cells embedded in or seeded on the interface of extracell matrix (ECM), or membranes situated next to perfusable microchannels that provide nutrients, waste clearance, and stimulate flow^19^. For studying drug toxicity, the Ingberr group established a ‘kidney-on-a-chip’ by using primary human kidney proximal tubular epithelial cells to observe *in vivo*-like pathophysiology^20^. In addition, the renal microfluidic environment is a key modulator of tubular pathological change. We established a renal tubule-on-a-chip lined by immortalized human proximal tubule cells to recreate the epithelial-to-mesenchymal transition that causes renal interstitial fibrosis during the development of proteinuric nephropathy^21^. Besides tubular reabsorption, another important function of the kidney is filtration performed by glomeruli. Glomerular disease is the most common kidney disease. Previous studies only focused on the construction of models of the renal tubular system. Microfluidic devices that simulate glomerular physiology and pathology using such chips have not been reported.

Here, we developed a multifunctional “glomerulus-on-a-chip” (GC) that reconstructs the pivotal structure of glomeruli and realizes glomerular filtration function in a physiological microenvironment with complex hemodynamic factors. Under the spatial and temporal control of hydrodynamic factors, this microfluidic device could develop into a model of hypertensive nephropathy. First, we investigated the effects of dynamic alterations on permeability of the glomerular filtration barrier (GFB; the fundamental functional unit of a glomerulus) and biological changes in glomerular cells. Alterations in cytoskeletal architecture and/or expression of intercellular junction proteins in glomeruli (e.g., nephrin, podocin) are present in patients with hypertensive nephropathy^22^,^23^, so we investigated the effects of dynamic alterations on the cytoskeletal architecture and expression of important proteins of glomerular cells on the GC. Then, we compared the results from the GC with normal and hypertensive rats to assess our GC. Overall, this GC, expands the available organ-level models of glomerular disease *in vitro*, is an alternative to conventional cell culture and animal models, and could be applied to research of the complex microenvironmental cues that regulate tissue injury and disease progression.

## Results

### Constitution of a functional GC and representations

**Figure 1:**
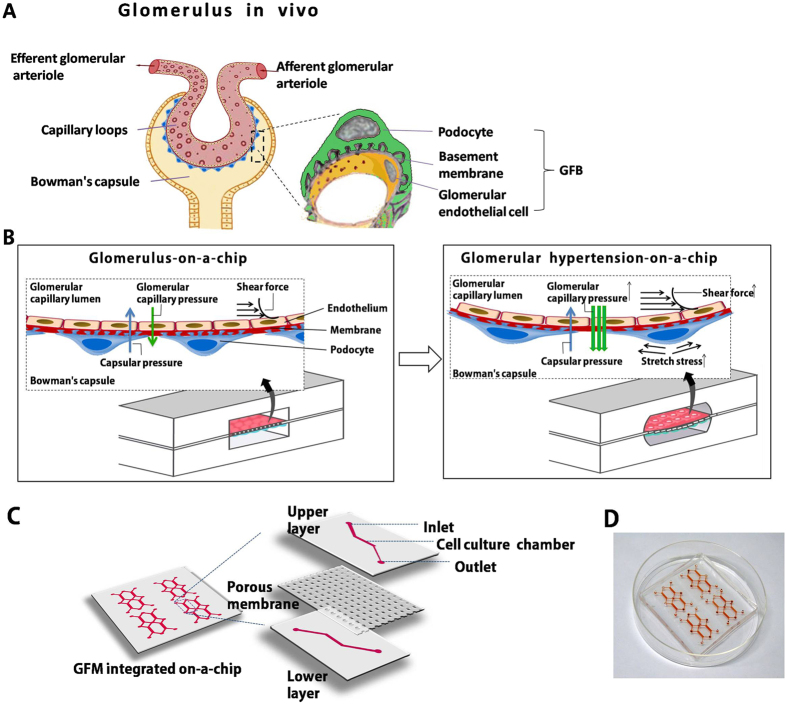
Biologically inspired design of a filtration functional glomerulus-on-a-chip (GC) microdevice. (**A**) When blood flows through afferent glomerular arterioles into the glomerular loops, the glomerular filtrate penetrates the GFB to the Bowman’s capsule driven by effective filtration pressure. The GFB comprised the glomerular endothelium, glomerular basement membrane and podocyte in sequence. (**B**) The effective filtration pressure depends on glomerular capillary pressure, capsular pressure and shear force. The microfabricated glomerulus mimic device uses compartmentalized PDMS microchannels to form a GFB on a membrane coated with ECM. (B1) The device recreated the physiological glomerular filtration function by supplying perfusion flow in the upper microchannel and causing mechanical forces (e.g., glomerular capillary pressure, shear force, and stretch stress) to act on the GFB membrane; (B2) a pathological glomerular microenvironment‍ was established by perfusion flow regulating mechanical forces. (**C**) Sixteen culture chambers integrated on-a-chip were composed of upper and lower PDMS layers and a middle polycarbonate layer. Three layers were aligned and irreversibly bonded to form two sets of microchannels separated by a porous membrane (10 μm thick) containing an array of through-holes with an effective diameter of 10 μm. (**D**) Image of the GC microfluidic device viewed from above.

A glomerulus is a repeatedly branched capillary network in which capillary loops formed by afferent glomerular arterioles converge on efferent glomerular arterioles (Fig. 1A). The intervening membrane was coated with an extract of the basement membrane, and mice glomerular endothelial cells (GEnCs) and mice podocytes (MPC-5) were cultured on opposite sides of the membrane (Fig. 1B). When cells had grown to confluence, a culture medium at a flow rate of 5 μL/min was introduced into the endothelial compartment through the inlets of the upper layer, and waste culture medium was collected from the podocyte compartment through the outlets of the lower layer. Hence, the microenvironment of the glomerulus was mimicked precisely (Fig. 1B1). To simulate the glomerular hypertensive microenvironment of hypertensive nephropathy in the GC, we perfused the culture medium at a flow-rate of 10 and 15 μL/min through the endothelial channel (Fig. 1B2). When the different perfusion flow rates of 5, 10 and 15 μL/min were applied to the microdevice, the average bottom wall shear stresses in the culture chambers of the upper layer were 0.001, 0.002 and 0.003 dyn/cm^2^, respectively. We established a GC with the same four units. Each unit contained four cell-culture chambers interconnected to simulate the network structure of glomerular capillary loops. The width of sample-in channels was wider than the width of sample-out channels because the extent of contraction of afferent glomerular arterioles is less than that of efferent glomerular arterioles^24^. Each unit was designed to connect with a common inlet that was linked to the syringe pump to apply a medium flow rate. The main functional structure of a glomerulus was constituted by two closely apposed microchannels separated by a thin (10 μm), porous, polyester carbonate membrane (Fig. 1C). We integrated four glomerular units on the same chip to improve experimental efficiency (Fig. 1C,D).

**Figure 2:**
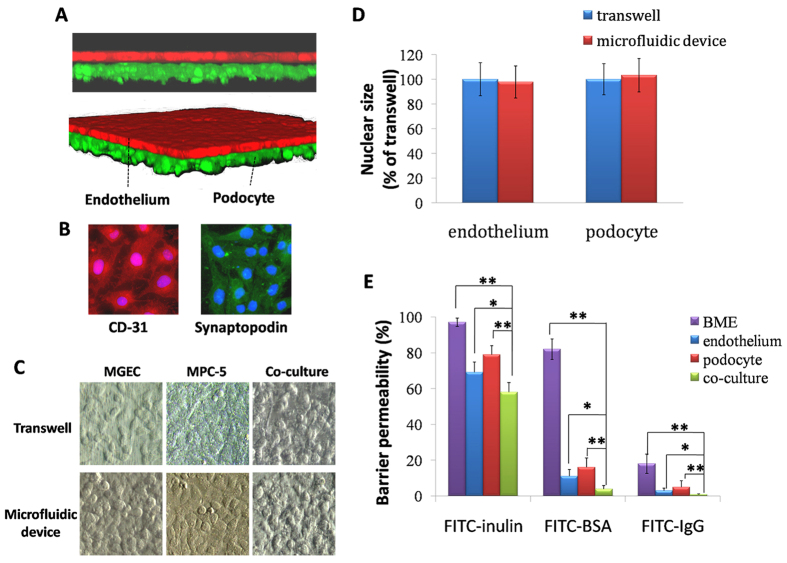
Rationale and applicability of the physiologic GC. (**A**) After 72 h of co-culture in the GC, a single layer of the glomerular endothelium (endothelium stained with CellTracker Red™, Thermo Scientific) closely apposed to a monolayer of podocytes (podocytes stained with CellTracker Green, Thermo Scientific), both of which were separated by a thin, extracellular membrane-coated membrane. (**B**) Immunostaining of endothelium CD-31 (red) and podocyte (green) synaptopodin after 3 days of co-culture. (**C**) Representative phase-contrast images do not show different morphology between cells in the transwell and cells in the GC. All images are x400 magnification. (**D**) Quantification of apoptosis in the endothelium and podocyte show no differences in the microfluidic device and in the transwell. (**E**) GFB permeabilities obtained by measuring the rate of transport of FITC-inulin, FITC-BSA and FITC-IgG are significantly reduced in co-cultures compared with BME-coated only and monocultures of the endothelium or podocytes under a perfusion flow rate of 5 μL/min. Data are the mean ± SD from three separate experiments. *P < 0.05, **P < 0.01.

Then, we investigated the function and feasibility of the GC. When GEnCs and MPC-5 cells were introduced into their respective channels, they became attached to opposite surfaces of the coating of the basement-membrane extract (BME), and formed intact monolayers (Fig. 2A). Normal expression of cellular biomarkers for endothelial cells and podocytes (CD-31 and synaptopodin) was observed in the GC (Fig. 2B). We found that cells grew within the GC (Fig. 2C), and that the rate of apoptosis in the GC and transwell was not significantly different (P > 0.05) (Fig. 2D). We used 1 mg/mL of fluorescein isothiocyanate (FITC)-conjugated inulin, FITC-bovine serum albumin (BSA), and FITC-IgG to approximate small-molecular weight (MW), medium-MW and large-MW substances in the blood, respectively. GFB permeability of the co-culture was significantly lower than that for the monoculture and only BME-coated membrane (P < 0.05). Permeability of FITC-inulin was ≈60%, for FITC-BSA it was ≈3%, and for FITC-IgG it was ≈0.1% (Fig. 2E).

### Mimicking hypertensive nephropathy in the GC

**Figure 3:**
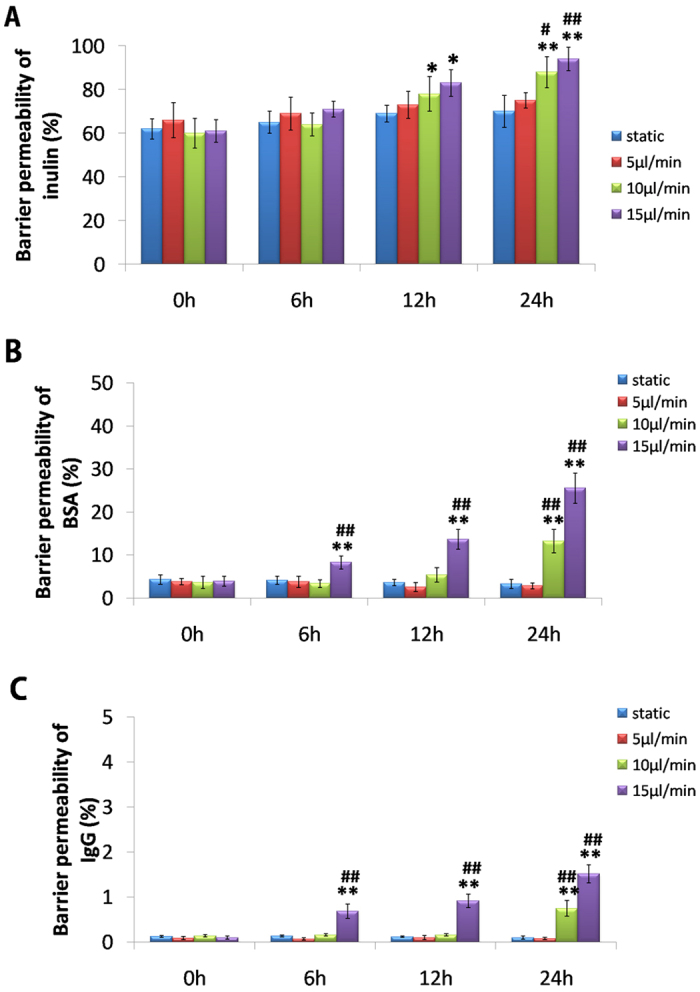
Quantitative analyses of GFB permeabilities in progression of hypertensive nephropathy on-a-chip. (**A**) Quantification of barrier permeability to FITC-inulin is shown. (**B**) Quantification of barrier permeability to FITC-BSA is shown. (**C**) Quantification of barrier permeability to FITC-IgG is shown. Data are the mean ± SD from three separate experiments. *P < 0.05, **P < 0.01 *vs*. static group; ^#^P < 0.05, ^##^P < 0.01 *vs*. 5 μL/min group.

To simulate the glomerular hypertensive microenvironment for hypertensive nephropathy in the GC, we perfused the culture medium at a flow-rate of 10 and 15 μL/min through the endothelium channel of the GC. Then, we examined filtration functions in different glomerular hemodynamic conditions by measuring the permeability of the microengineered GFB to FITC-inulin, FITC-BSA and FITC-IgG that had been added into the endothelium channel. Fluorescence intensity of fluids collected from the podocyte compartment was monitored 6, 12 and 24 h after endothelial exposure to different hemodynamic conditions. When GFB were exposed to a perfusion rate of 5 μL/min (which mimicked physiological glomerular hemodynamics), the GFB permeability of inulin, BSA and IgG did not change (Fig. 3). However, when the GFB was subjected to a perfusion rate of 10 and 15 μL/min (which mimics glomerular hypertension in hypertensive nephropathy), an increase in the permeability of inulin was detected from 12 h (P < 0.05) (Fig. 3A); also, the permeability of BSA and IgG increased significantly from 6 h (P < 0.01) under a perfusion rate of 15 μL/min, whereas an increase at 24 h was observed at 10 μL/min (Fig. 3B,C).

### Pathological injury to the glomerular endothelium due to glomerular hypertension

**Figure 4:**
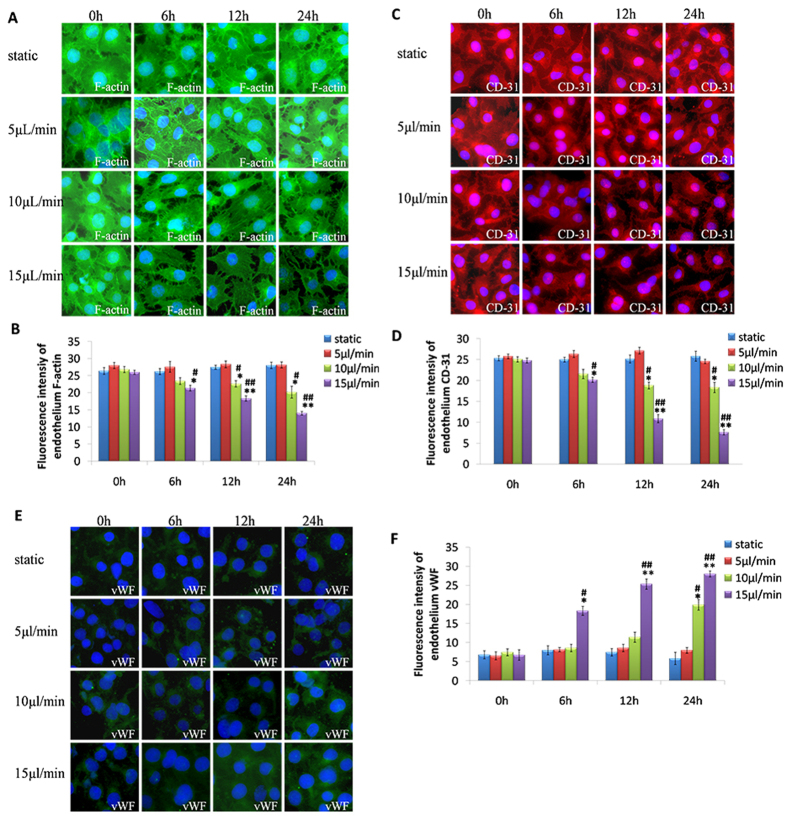
Hyper-perfusion damages the glomerular endothelium. (**A**) Immunostaining of endothelial cytoskeletal F-actin after exposure to a perfusion flow rate of 5, 10 and 15 μL/min, respectively, through the endothelium channel of the microdevice for 0, 6, 12 and 24 h. (**B**) Quantification of fluorescence intensity is also shown for F-actin. (**C**) Immunostaining of endothelial CD-31 in cell–cell junctions after exposure to a perfusion flow rate of 5, 10 and 15 μL/min, respectively, through the endothelium channel of the microdevice for 0, 6, 12 and 24 h. (**D**) Quantification of fluorescence intensity is also shown for CD-31. (**E**) Immunostaining of endothelial vWF after exposure to a perfusion flow rate of 5, 10 and 15 μL/min, respectively, through the endothelium channel of the microdevice for 0, 6, 12 and 24 h. (**F**) Quantification of fluorescent intensity is also shown for vWF. All images are at x400 magnification. Data are the mean ± SD from three separate experiments. *P < 0.05, **P < 0.01 *vs*. static group; ^#^P < 0.05, ^##^P < 0.01 *vs*. 5 μL/min group.

To understand the mechanism of increased glomerular filtration of medium-MW and large-MW material caused by glomerular hypertension, we tested further the effects of glomerular hypertension on the endothelium and podocytes. Cytoskeletons of glomerular endothelial cells comprise mainly F-actin and affect the morphology and contractility of cells directly^25^,^26^,^27^. Our data showed that GEnCs in the static culture were polygonal and had few F-actin microfilaments, which traversed the cell body at random and in multiple directions (though some stress fibers were at the cell periphery). More stress fibers (instead of microfilaments) were observed in the cell body when a perfusion rate of 5 μL/min induced GEnCs. With increasing perfusion rate and prolongation of incubation time, more regular stress fibers in the cell center were observed (10 μL/min perfusion rate); also, the structure of the cell periphery changed from filopodia to lamellipodia, and cellular shape became irregular (15 μL/min perfusion rate) (Fig. 4A). Analyses of fluorescence intensity showed reduced expression of F-actin in the endothelium in the 10 μL/min perfusion rate-treated group after 12 h and 15 μL/min perfusion rate-treated group after 6 h (Fig. 4B).

CD-31 has a key role in intercellular junctions and adhesion to the endothelium. CD-31 immunostaining revealed that a perfusion rate of 5 μL/min on GEnCs for 24 h had no effect on CD-31 expression. However, CD-31 expression showed time- and perfusion rate-dependent changes upon increasing perfusion rate (Fig. 4C,D). To further study injury to GEnCs, we explored expression of a classic biomarker of damage to the vascular endothelium: von Willebrand factor (vWF)^28^. Under static and 5 μl/min conditions, vWF expression was limited to the cytoplasm surrounding the nucleus. However, under the hyperperfusion conditions of 10 and 15 μL/min, vWF expression showed time- and rate-dependent increases (Fig. 4E,F).

### Pathological injury to podocytes due to glomerular hypertension

**Figure 5:**
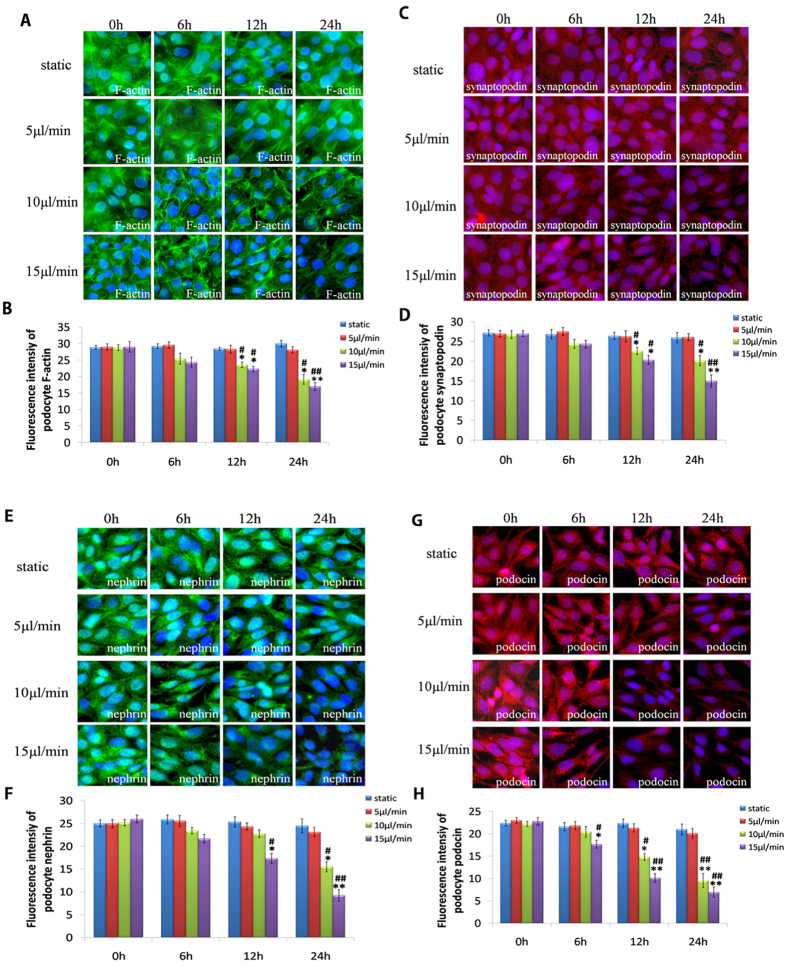
Hyper-perfusion damages glomerular podocytes. (**A**) Immunostaining of podocyte cytoskeletal F-actin after exposure to a perfusion flow rate of 5, 10 and 15 μL/min, respectively, through the endothelium channel of the microdevice for 0, 6, 12 and 24 h. (**B**) Quantification of fluorescence intensity is also shown for F-actin. (**C**) Immunostaining of podocyte cytoskeletal synaptopodin after exposure to a perfusion flow rate of 5, 10 and 15 μL/min, respectively, through the endothelium channel of the microdevice for 0, 6, 12 and 24 h. (**D**) Quantification of fluorescence intensity is also shown for synaptopodin. (**E**) Immunostaining of podocyte nephrin after exposure to a perfusion flow rate of 5, 10 and 15 μL/min, respectively, through the endothelium channel of the microdevice for 0, 6, 12 and 24 h. (**F**) Quantification of fluorescence intensity is also shown for nephrin. (**G**) Immunostaining of podocyte podocin after exposure to a perfusion flow rate of 5, 10 and 15 μL/min, respectively, through the endothelium channel of the microdevice for 0, 6, 12 and 24 h. (**H**) Quantification of fluorescence intensity is also shown for podocin. All images are at x400 magnification. Data are the mean ± SD from three separate experiments. *P < 0.05, **P < 0.01 *vs*. static group; ^#^P < 0.05, ^##^P < 0.01 *vs*. 5 μL/min group.

Using GCs, we explored the effects of glomerular hypertension on the cytoskeletal organization of F-actin and synaptopodin, and slit diaphragm proteins (nephrin and podocin) of MPC-5 cells. Our data showed that F-actin organization was changed severely under the condition of fluidic flow. Strong microfilaments were found to diffuse though the cytoplasm in multiple directions in static podocytes. After 5 μL/min perfusion, strong microfilaments exhibited a regular and directional distribution in cytoplasm. However, under hyperperfusion (10 and 15 μL/min), expression of F-actin transferred from the cytoplasm to the cell periphery (Fig. 5A). A significant reduction in F-actin expression was observed in 10 and 15 μL/min groups (P < 0.05) (Fig. 5B).

Synaptopodin is an actin-associated protein that may have roles in actin-associated cell shape and motility^29^. Consistent with changes in F-actin expression, immunofluorescence examination of MPC-5 cells in static culture showed a linear conformation of synaptopodin distributed uniformly in the cytoplasm. Synaptopodin realigned in the direction of flow upon perfusion at 5 μL/min for 24 h. Increasing the perfusion rate to 10 and 15 μL/min led to perfusion rate- and time-dependent morphological changes in MPC-5 cells: they changed from a polygonal form to a spindle, fibroblast-like form (Fig. 5C). Immunofluorescence results showed reduced expression of synaptopodin in hyperperfusion-treated groups (Fig. 5C,D).

Recent studies have shown that the cytoskeleton of foot processes (especially slit diaphragm proteins in podocytes) may have an important role in maintenance of GFB function in patients with nephropathy^30^. Hence, we explored expression of nephrin and podocin that most likely determine the mechanical properties of slit diaphragm proteins^31^. Induction of hyperperfusion reduced expression of nephrin and podocin in a time- and perfusion rate-dependent manner (Fig. 5E–H).

### Pathological effects of glomerular perfusion on gap areas between cells

We have demonstrated that hyperperfusion has detrimental effects upon GFB permeability in the GC, primarily due to pathological changes in the actin cytoskeleton and intercellular junctions in the glomerular endothelium and podocytes (Figs 4 and 5). Immunostaining analyses of intercellular junctions revealed that a hyperperfusion rate (10 and 15 μL/min) resulted in an increased cell-cell gap area in endothelial and podocyte monolayers relative to controls (Supplementary Figure 1A,B).

### Comparison of GCs with hypertensive nephropathic rats

**Figure 6:**
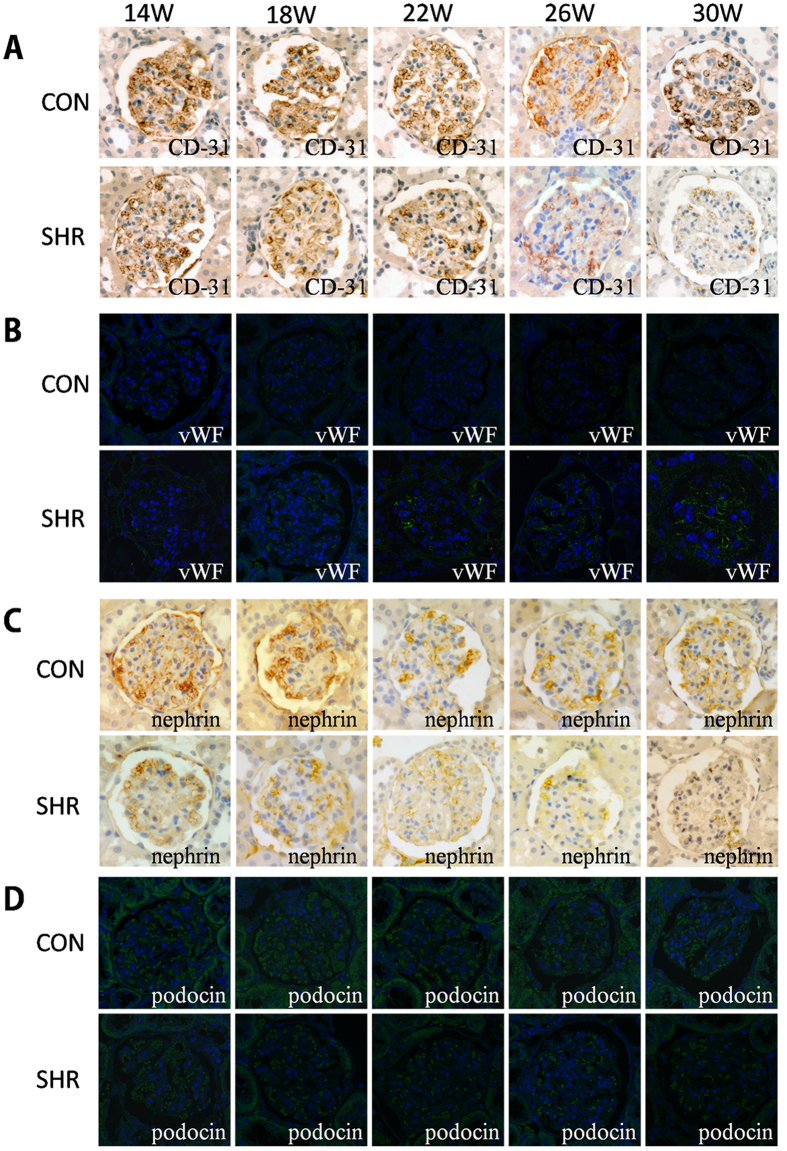
Pathologic changes in CD-31, vWF, nephrin and podocin in SHRs. (**A**) Immunohistochemistry showed reduced expression of the endothelial marker CD-31 in the SHR group, but not in the glomerulus of the control group. (**B**) Immunofluorescence showed increased expression of the marker of endothelial injury, vWF, in the SHR group, but not in the glomerulus of control group. (**C**) Immunohistochemistry showed reduced expression of the podocyte marker nephrin in the SHR group, but not in the glomerulus of the control group. (**D**) Immunofluorescence showed reduced expression of the podocyte marker podocin in the SHR group, but not in the glomerulus of the control group. All images are at x400 magnification.

To investigate further the reliability of the physiological and pathological results mentioned above, we conducted similar studies in spontaneously hypertensive rats (SHRs). Mean blood pressure of each age (in weeks) of SHRs was significantly higher than that in the control group (P < 0.01) (Supplementary Figure 2A), a result that was consistent with the development of glomerulosclerosis in SHRs in a week age-dependent manner (Supplementary Figure 2B,C). Serum levels of creatinine and the creatinine clearance rate were not significantly different (P > 0.05) between SHRs and the control group, whereas the excretion rate of urinary protein increased with increasing week age in SHRs (P > 0.05) (Supplementary Table 1). Then, we measured the components of urinary protein. With increasing week age, concentrations of β2 microglobulin, albumin and IgG, representing small-MW proteins, medium-MW and large-MW proteins in urine, respectively, were increased significantly in SHRs compared with the control group (P < 0.001) (Table 1). Importantly, urinary excretion of proteins involved mainly medium-MW and small-MW proteins, which was consistent with observations of permeability damage induced by hyperperfusion in the GCs. CD-31 expression in the glomerular endothelium decreased with increasing week age in SHRs, whereas vWF expression increased. In glomerular podocytes, expression of nephrin and podocin decreased in a week age-dependent manner in SHRs (Fig. 6). These results confirmed that glomerular hypertension damaged the endothelium and intercellular junction structures in glomeruli.

## Discussion

Glomerular hemodynamics are dependent upon integrated mechanical forces (including filtration pressure, flow shear force, and traction force). We developed a multifunctional microdevice that reproduces the key structural, functional, and hemodynamic properties of the glomerulus (Figs 1 and 2). The compartmentalized channels of the device simulate the structure of the glomerular capillary lumen and Bowman’s capsule. We manipulated hemodynamic changes in glomerular capillaries by connecting a microsyringe pump to the GC and by accurate adjustment of the rate of perfusion of the culture medium to the endothelium.

Endothelial cells and podocytes in the GC formed two cellular layers separated by a thin, ECM-coated membrane, which constituted the structure of a glomerulus (Fig. 2A). We found that this arrangement could simulate the hemodynamic microenvironment in the glomerulus in physiological conditions when we used a flow rate of 5 μL/min in endothelial channels. Cell growth was good, as was biomarker expression (Fig. 2B–D). The GC could filter most inulin molecules, but few molecules of albumin and IgG (Fig. 2E). These data suggested that the structure and filtration function of GC were high, in accordance with those in the human kidney *in vivo*^32^.

In hypertensive nephropathy, glomerular hemodynamics undergo pathological changes at high perfusion pressure, high filtration pressure, and high transmembrane pressure, which injure glomerular filtration function^3^. We simulated the glomerular microenvironment of hypertensive nephropathy using the GC by increasing perfusion flow rates in endothelial channels. Because of the *in vivo*-like structure of the GC, increasing perfusion flow rates in the upper microchannel induced increased mechanical forces in glomeruli (e.g., glomerular capillary pressure, shear force, and stretch stress) (Fig. 1B2). Meanwhile, the narrowness of the outflow channel of endothelial cells increased the transmembrane pressure acting on the GFB in the GC, which simulates the glomerular microenvironment *in vivo* when hypertension causes renal damage (Fig. 1C). Then, we detected glomerular filtration function under hyperperfusion conditions. When the GC was exposed to a flow rate of 10 μL/min, filtration of inulin, albumin, and IgG increased at 24 h (Fig. 3). Importantly, the increasing degree of filtration of BSA (20%) was much larger than that of IgG (1%) (Fig. 3B,C). These results were consistent with the clinical manifestations of hypertensive nephropathy: increased excretion of small-MW proteins and albumin, rather than large-MW IgG, in urine^33^. Our data provide direct evidence that an integrated mechanical force causes glomerular injury.

Regulation of endothelial cells for vascular permeability is dependent mainly on cytoskeletal proteins and intercellular connections^34^. Therefore, we investigated changes in classical cytoskeletal proteins, intercellular connections, and damage markers of endothelial cells under pathological mechanical forces. Our results demonstrated that, if endothelial cells were stimulated by perfusion under pathological conditions, the cytoskeleton of F-actin became rearranged, connections between cells became loosened, and cells contracted. As the perfusion rate and time increased, F-actin expression decreased (Fig. 4A,B). These results suggest that hemodynamic factors in the glomerulus can cause cytoskeletal rearrangement in endothelial cells, cell contraction, and changes in connections between cells, thereby affecting the filtration function of the glomerulus.

Recent studies have demonstrated that cognate binding of CD-31 can contribute to the stability of connections between endothelial cells^35^. Our results demonstrated that, as the perfusion rate and time increased in endothelial channels, CD-31 expression decreased (Fig. 4C,D). These data suggest that glomerular hypertension could damage intercellular junctions of the endothelium, which causes injury to glomerular barrier function.

vWF is a classical marker of injury to endothelial cells. Studies have demonstrated that, compared with healthy subjects, serum levels of vWF in CKD patients with proteinuria are significantly higher^36^. Our study showed that, upon stimulation by low perfusion (5 μL/min), the distribution and expression of vWF was not significantly different to that of the static culture group. As the perfusion rate increased, increased expression of vWF was observed (Fig. 4E,F).

Podocytes are the morphological basis for high hydraulic transmission in glomerular capillaries, which is responsible for the selective filtering function of the glomerulus^37^. We investigated the effects of glomerular hypertension on podocytes using the GC. As the leading constituent of the cytoskeleton, F-actin is the foundation that maintains podocyte morphology. Synaptopodin is a linear cytoskeletal protein connected to F-actin. With increasing perfusion pressure in the endothelium channel and time, F-actin expression in podocytes decreased gradually (Fig. 5A,B), which corresponded to changes in synaptopodin expression (Fig. 5C,D). Downregulation of the podocyte actin cytoskeleton has been observed in hypertensive-nephropathy patients with proteinuria^38^. Our results reproduced this pathological process vividly and provided direct evidence that abnormal mechanical forces induced reduced expression of F-actin and synaptopodin in podocytes. This phenomenon might be responsible for proteinuria in hypertensive nephropathy.

Nephrin and podocin are major proteins on the slit membranes of podocytes. Slit membranes are the basic structures of podocytes that carry out selective filtering in glomerular capillaries^37^. With increasing perfusion pressure and prolonged duration of increased perfusion pressure, we found that high perfusion pressure and high transmembrane pressure within the glomerulus caused a reduction in expression of nephrin and podocin (Fig. 5E–H).

Hence, our results suggested that hypertensive hemodynamic factors in glomeruli damaged cytoskeletal proteins and intercellular junctions directly. Abnormal hemodynamics caused increased gap areas between endothelial cells and podocytes (Supplementary Figure 1) and damaged the GFB, which results in proteinuria in patients with hypertensive nephropathy.

Finally, we used SHRs to verify the feasibility of the GC. Increases in mean blood pressure and the degree of glomerulosclerosis were observed in SHR groups with increasing age. Our data showed that glomerular injury could be caused by hypertension in SHRs (Supplementary Figure 2). Urinary concentrations of microalbumin in SHRs were significantly higher than those in the control group with increasing week age. Major components of proteinuria were β2-microglobulin and albumin (96.6%) rather than IgG (3.4%) (Table 1). These data were consistent with our results in the GC (Figs 2 and 3). Then, we measured the classical markers of endothelial cells and podocytes in rat kidneys. Compared with the control group, CD31 expression was obviously downregulated and expression of the injury marker vWF was upregulated in SHRs. Reduced expression of nephrin and podocin in glomeruli were also observed with increasing week age in SHRs (Fig. 6). These results were consistent with the data obtained in the GC (Figs 4 and 5).

With the exception of the glomerular endothelium and podocytes that constitute the GFB, mesangial cells in the glomerulus have a role in supporting glomerular capillaries. Mesangial cells in the glomerulus could conduct immune function change. We did not add mesangial cells into the GC, so some kidney diseases of mesangial cell injury originating from inflammation of the glomeruli (e.g., mesangial proliferative glomerulonephritis) could not be simulated in this GC.

In summary, we created a GC upon which the basic physiological structure and function of the glomerulus could be reproduced. This GC can be used to detect changes in cellular morphology and protein expression. It can also be developed as a model of hypertensive nephropathy by adjustment of the flow rate and type of nutrient solution employed. Endothelial cells and podocytes are crucial targets in various glomerular diseases, so this GC could provide a potential technical platform for detection of the sensitivity and toxicity of drugs (e.g., glucocorticoids, immunosuppressants) for patients with glomerular disease.

## Methods

### Design and fabrication of the GC

A schematic of the GC is shown in Fig. 1B. The GC comprised three layers, in which the middle layer was a porous polycarbonate membrane coating BME (Cultrex™ Basement Membrane Extract; R&D Systems, Minneapolis, MN, USA). The major components of the BME included laminin, collagen IV, entactin, and heparin sulfate proteoglycan, which was approximate to that of the glomerular basement membrane. The upper and lower polydimethylsiloxane (PDMS) layers contained four units. Each unit contained four cell-culture chambers fabricated in PDMS using a rapid-prototyping method^21^, and connected to an inflow channel and outflow channel. Channels in the upper layer mimicked glomerular capillaries: the inflow channel mimicked afferent glomerular arterioles and the outflow channel mimicked efferent glomerular arterioles. The degree of contraction of efferent glomerular arterioles is greater than that of afferent glomerular arterioles (Fig. 1A), so the width of the outflow channel was 300 μm, much less than that of the inflow channel (1000 μm) (Fig. 1B). Channels in the lower layer mimicked Bowman’s capsule. Each unit was designed to connect with a common inlet linked to a syringe pump for controlling the flow of the culture medium, and an outlet for collecting waste medium. The flow rate was set at 5, 10 and 15 μL/min separately to simulate physiologic and pathologic hemodynamic microenvironments. Testing of the leakage features of all channels was carried out using red ink, and demonstrated no interference or mixing between culture chambers (Fig. 1D). Each cell-culture chamber was designed to have a height of 100 μm, width of 1000 μm, and length of 10^4^ μm. The finished GC was sterilized with ultraviolet light for 30 min before use.

### Cell culture and co-culture

The immortalized GEnCs line, a gift from Chuanming Hao at Huashan Hospital (affiliated with Fudan University, Shanghai, China), was cultured in RPMI-1640 medium supplemented with 10% fetal bovine serum (FBS; Thermo Scientific, Waltham, MA, USA), 100 U/mL penicillin and 100 μg/mL streptomycin at 37 °C in an atmosphere of 5% CO_2_. Immortalized MPC5 cells were kindly provided by Xiangmei Chen (Chinese PLA General Hospital, Beijing, China). Undifferentiated MPC5 cells were cultured in RPMI-1640 medium supplemented with 10% FBS (Thermo Scientific), 100 U/mL penicillin, 100 μg/mL streptomycin and 10 U/mL recombinant mouse interferon-γ (PeproTech, Rocky Hill, NJ, USA) at 33 °C for proliferation. Cells were differentiated at 37 °C under interferon-γ depletion for 10–14 days^39^.

To establish a co-culture microsystem, a GEnCs suspension was loaded into the upper chamber of the GC through a common reservoir. After GEnCs became adherent, MPC5 cells were seeded in the lower chamber. After cells became confluent, the cell suspension was loaded into GC through a common reservoir.

### Flow experiments

After cell adherence, they were exposed to a perfusion flow rate of 5, 10 and 15 μL/min for 6, 12, and 24 h with RPMI-1640 medium using a syringe pump (KD Scientific, Holliston, MA, USA) at 37 °C. Before and during device loading, prepared samples were mixed to achieve uniform cell density and set the appropriate perfusion flow rate. The waste culture medium was collected alternate days.

To evaluate the local fluid shear stress distribution in the chamber, the flow was assumed to be laminar, viscous, and incompressible. We designed the microfluidic networks based on the electric circuit analogy^40^. Using this analogy, individual channel sections were treated as resistances within the flow circuit. We used an approximate equation to model this system:


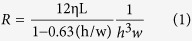


where R is the hydraulic resistance, *η* is the fluid viscosity, *w* is the channel width, and *h* is the channel height, for h < w^41^. For a square microchannel, the resistance can be calculated by


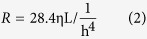


A constant pressure drop Δ*p* results in a constant flow rate Q. This result can be summarized using the Hagen–Poiseuille equation, as follows:


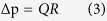


The density of the perfusion medium was 993.2 kg/m^3^, and its viscosity was 7.85 × 10^−4 ^Pa s at 37 °C^42^.

### Immunofluorescence staining

Expression of CD-31, F-actin, and vWF in GEnCs, as well as synaptopodin, F-actin, nephrin and podocin in MPC-5 cells, was done using an immunofluorescence assay. After treatment for different times, GEnCs or MPC-5 cells on the bottom layer were washed thrice in phosphate-buffered saline (PBS) and fixed in 4% paraformaldehyde for 15 min at room temperature. After washing thrice in PBS, nonspecific hybridization was blocked with goat serum for 1 h at room temperature. Cells were incubated separately with monoclonal rabbit anti-mouse CD-31, (Abcam, Cambridge, UK), monoclonal mouse anti-mouse synaptopodin (Abcam), and monoclonal rabbit anti-mouse nephrin and podocin (Abcam) primary antibody at a 1:100 dilution overnight. The next day, cells were rinsed thrice with PBS (5-min each), and incubated with the appropriate secondary antibody at a dilution of 1:100 for 1 h. Expression of F-actin and vWF were measured by direct immunofluorescence staining. After pretreatment, cells were incubated with phalloidin-FITC (0.5 mg/mL; Sigma–Aldrich) or FITC-vWF antibody (1:100 dilution; Abcam) for 2 h at 37 °C.

After washing in PBS, the GC was mounted with antifade reagent (Fluoromount-G; Southern Biotech, Birmingham, AL, USA) and placed under a coverslip. Digital images were captured using an inverted fluorescence microscope.

### Permeability assay

Permeability of the GFB was assessed by measuring the rate of transport of FITC-inulin, FITC-BSA and FITC-IgG from the upper endothelial channel to the lower podocyte compartment. FITC-inulin (1 mg/mL in RPMI-1640 medium) was introduced into the upper channel; transport of inulin, BSA or IgG across the barrier was determined by serial sampling of fluid from the lower channel and measurement of its fluorescence intensity (which was used as an index of permeability of the GFB).

### Care and use of laboratory animals

Animal experiments were conducted in accordance with the regulations set by the Institutional Committee for the care and use of Laboratory Animals, and approved by Dalian Medical University Laboratory Animal Center. Adult male Wistar Kyoto rats and SHR (320–460 g) were housed in cages and exposed to a 12 h light–dark cycle. They had free access to food and water. Rats were killed at weeks 14, 18, 22, 26, and 30 (n = 6 at each time point). Kidneys were harvested for morphologic and biochemical studies.

### Measurement of protein concentration by enzyme-linked immunosorbent assay (ELISA)

Concentrations of β2-microglobulin, microalbumin, and IgG in rat urine were determined using an ELISA kit (Elabscience Biotechnology, Wuhan, China). Samples were diluted two- to fourfold with homogenization buffer before analyses. Assay results were normalized to those of the total protein concentrations obtained by the bicinchoninic acid assay.

### Immunohistochemical analyses of CD-31 and nephrin

First, specimens were fixed with formalin, embedded in paraffin, sectioned (thickness, 4 mm) and transferred to microscope slides. Sections were dewaxed in xylene followed by rehydration in a graded series of ethanol solutions. Then, they were incubated in citrate buffer (pH 6.0) and heated to 121 °C in an autoclave to retrieve the antigen. Endogenous peroxidase was inactivated after immersion into 0.3% hydrogen peroxide for 30 min. To eliminate nonspecific binding, specimens were incubated in 10% goat serum for 1 h at room temperature. Subsequently, sections were incubated with primary antibody: anti-CD-31 antibody (1:100; Abcam) and anti-nephrin antibody (1:100; Abcam) overnight. Immunoreactive staining was processed using the peroxidase–anti-peroxidase method according to manufacturer instructions (DAKO, Hamburg, Germany). 3, 39-diaminobenzidine tetrachloride (DAB) chromogen solution was used to detect the reaction. After rinsing in water for 30 min, sections were counterstained with hematoxylin, and dehydrated. Finally, they were mounted in mounting medium for interpretation. For all antibodies, negative controls were used in which the primary antibody was omitted. All sections were negative.

### Immunohistofluorescence analyses of vWF and podocin

Cryostat sections (thickness, 4 mm) fixed in cold acetone were incubated with FITC-vWF (Abcam) antibody and rabbit anti-podocin overnight, and then the next day with FITC-labeled anti-rabbit IgG (Biosource, Camarillo, CA, USA) for 1 h at 37 °C in the dark. After washing thrice with PBS for 3 min, sections were counterstained with 1 mg/mL 40′,6-diamidino-2-phenylindole for 1 min and mounted in anti-fade medium. Slides were viewed under a confocal microscope (TCS-SL; Leica, Bannockburn, IL, USA). Staining intensity was analyzed using image-analysis software (GraphPad, San Diego, CA, USA).

### Statistical analyses

Data are the mean ± SD. For immunofluorescence analyses, quantitation was done by scanning and analyzing the intensity of the fluorescence using Image-Pro Plus v6.0 (Media Cybernetics, Silver Spring, MD, USA). Statistical analyses of data were done by Student’s *t*-test using SPSS v13.0 (IBM, Armonk, NY, USA). P < 0.05 was considered significant.

## Additional Information

**How to cite this article**: Zhou, M. *et al*. Development of a Functional Glomerulus at the Organ Level on a Chip to Mimic Hypertensive Nephropathy. *Sci. Rep.* **6**, 31771; doi: 10.1038/srep31771 (2016).

## Supplementary Material

Supplementary Information

## Figures and Tables

**Table 1:** Urinary concentrations of β2-microglobulin, microalbumin and IgG in each control and SHR group (x ± s).

Week age	Groups	Urineβ2 microglobulin (pg/L)	Urine albumin (mg/L)	Urine IgG (mg/L)
14	CON	42 ± 8.37	14.33 ± 5.45	0.26 ± 0.08
	SHR	76 ± 14.9*	68.83 ± 10.53^††^	0.98 ± 0.22^##^
18	CON	51 ± 10.42	19.79 ± 7.55	0.31 ± 0.09
	SHR	88 ± 16.50*	76.50 ± 15.05^††^	1.44 ± 0.55^##^
22	CON	53 ± 10.10	18.96 ± 8.58	0.32 ± 0.10
	SHR	102.50 ± 25.50**	109.20 ± 16.97^†††^	3.07 ± 0.90^###^
26	CON	52 ± 10.51	13.55 ± 7.78	0.33 ± 0.11
	SHR	117 ± 28.21**	150.67 ± 7.09^†††^	7.20 ± 1.13^###^
30	CON	46 ± 7.50	18.50 ± 8.26	0.35 ± 0.11
	SHR	132 ± 27.36**	181.60 ± 15.68^†††^	10.70 ± 1.96^###^

*P < 0.05, **P < 0.01 vs. urinary β2 microglobulin of the control group at each week age, ^†^P < 0.05, ^††^P < 0.01, ^††^P < 0.001 vs. urinary albumin of the control group at each week age, ^#^P < 0.05, ^##^P < 0.01, ^###^P < 0.001 vs. urinary IgG of the control group at each week age.
