# Supplementary material for: Development of a Functional Glomerulus at the Organ Level on a Chip to Mimic Hypertensive Nephropathy
Source: Sci Rep. 2016 Aug 25;6:31771. doi: 10.1038/srep31771 (PMC4997336; doi:10.1038/srep31771)
Supplement: Supplementary Information [file srep31771-s1.pdf]

# Development of a Functional Glomerulus at the Organ Level on a Chip to Mimic Hypertensive Nephropathy

Mengying Zhou[1†], Xulang Zhang[2†], Xinyu Wen[1], Taihua Wu[1], Weidong Wang[1], Mingzhou Yang[3], JingWang[1], MingFang[1], Bingcheng Lin[2]*, Hongli Lin[1]*

1 Department of Nephrology, The First Affiliated Hospital of Dalian Medical University, Key Laboratory of Kidney Disease of Liaoning Province, The Center for the Transformation Medicine of Kidney Disease of Liaoning Province, No. 222 Zhongshan Road, Dalian 116011, China.

2 Department of Biotechnology, Dalian Institute of Chemical Physics, Chinese Academy of Sciences, No.457 Zhongshan Road, Dalian 116023, China

3 Department of Urology, Dalian Friendship Hospital, No. 8 Sanba Square, Dalian, 116001, China

† These authors contributed equally to this work and share the first authorship.

* Corresponding author:

Hongli Lin, MD, PhD, Department of Nephrology, the First Affiliated Hospital of Dalian Medical University, No. 222 Zhongshan Road, Dalian 116011, China

Tel.: +86-411-83635963-3007

Fax: +86-411-83633689

E-mail: hllin@dlmedu.edu.cn

*Co-Corresponding author:

Bingcheng Lin, PhD, Department of Biotechnology, Dalian Institute of Chemical Physics, CAS, No.457 Zhongshan Road, Dalian 116023, China

Tel.: +86-411-8437-9065

Fax: +86-411-8437-9065

E-mail: bclin@dicp.ac.cn

**Supplementary Materials**

**Supplementary Methods**

**Apoptosis assay**

Nuclear size and the intensity of immunofluorescence staining by DAPI (Roche, Basel, Switzerland) can reflect the extent of apoptosis[43,44]. After treatment at different times, GEnCSs or MPC-5 cells on the bottom layer were washed thrice in PBS and fixed in 4% paraformaldehyde for 15 min at room temperature. After washing thrice in PBS, cells were incubated with DAPI for 15 min at room temperature. After washing thrice in PBS (5-min each), the GC was mounted with Fluoromount-G and placed under a coverslip. Digital images were captured using an inverted fluorescent microscope.

**Measurement of blood pressure in rats**

Systolic blood pressure (SBP) and diastolic blood pressure (DBP) of rats while special diets were eaten was measured by the tail-cuff method using Heater Scanner LE 5650/6 and Storage Pressure Meter LE 5002 (China–Japan Friendship Institute of Clinical Medical Science, Beijing, China). Rats were placed on a chamber preheated to 37 ℃. A clamp-type transducer and cuff were set up on their tails. SBP and DBP was measured at 37 ℃ for 10 min. Rats were trained in an acrylic restrainer every day for 1 week before BP measurements. SBP and DBP was measured when rats were unperturbed in the chamber during inflation–deflation cycles.

**Histology**

For histology, kidney tissues were fixed in 4% buffered paraformaldehyde and embedded in paraffin. Sections (thickness, 3 mm) were prepared and stained with periodic acid–Schiff.

To obtain the Glomerular Sclerosis Index (GSI), five slices was taken from each specimen, and then 30 glomeruli taken randomly from each section under light microscopy (×400 magnification). Image-Pro Plus v 6.0 (Media Cybernetics,) was used to calculate the percentage lesion area of the total glomerular area. Grading of the GSI was: 0: normal; 1: < 25.0%; 2: 25.0–50.0%; 3: 50.0–75.0%; 4: > 75.0%. The

mean integral from each section was taken, and a meal value within groups calculated.

**Biochemical indicators in blood and urine**

An automated clinical chemistry analyzer (Molecular Devices, Sunnyvale, CA, USA) was used for measurement of creatinine levels in serum, and protein levels in urine.

**Supplementary Figures:**

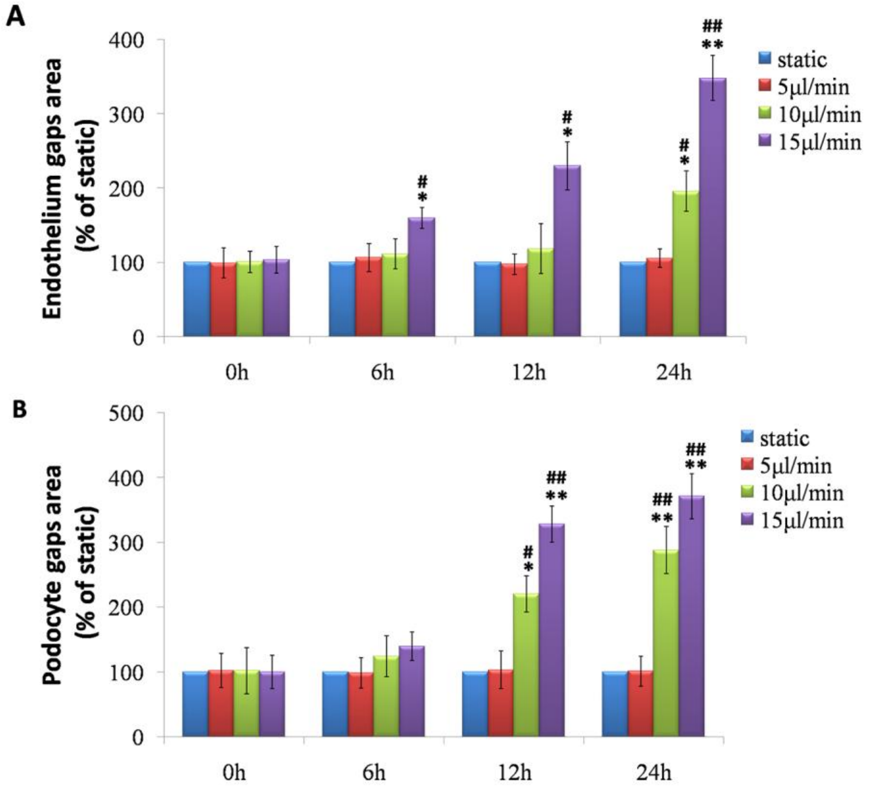


**Supplementary Figure 1.** Hyper-perfusion causes increases in areas of intercellular gaps. (A) Areas of gaps between endothelial cells is shown. (B) Areas of gaps between podocytes is shown. Data are the mean ± SD from three separate experiments. $^{*}$P<0.05, $^{**}$P<0.01 *vs*. perfusion flow rate of 10 μL/min; $^{*}$P < 0.05, $^{**}$P < 0.01 *vs*. static group; $^{\#}$P < 0.05, $^{\#\#}$P < 0.01 *vs*. 5 μL/min group.

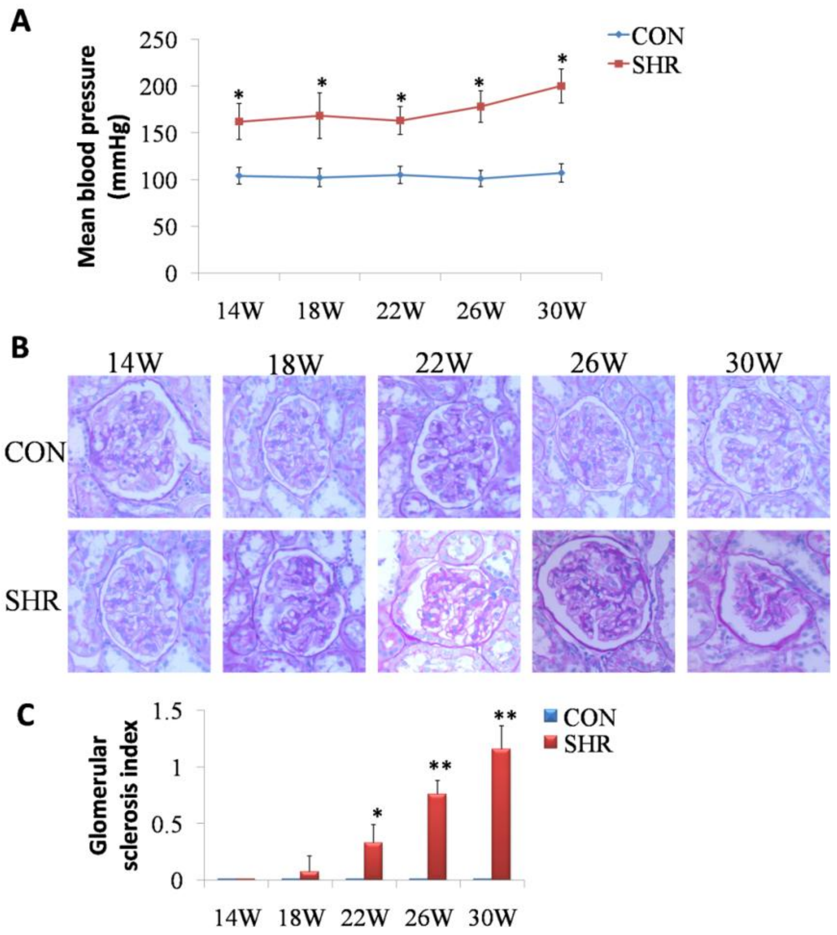


**Supplementary Figure 2.** Renal injury caused by spontaneous hypertension. (A) Mean blood pressure of different week age rats in control and SHR groups is shown. (B) Renal PAS staining of different week age rats in control and SHR groups is shown. (C) Quantification of glomerular sclerosis of rats in control and SHR groups at different week ages is shown. Data are the mean ± SD from three separate experiments. $^{*}P < 0.05$, $^{**}P < 0.01$ *vs*. the control group.

**Supplementary Table 1.** Body weight, renal function, and urinary excretion rate of protein in each control and SHRs group. ($\bar{x}\pm s$)

| Week age | Group | Body weight (g) | Serum creatinine (μmol/L) | Endogenous creatinine clearance rate (ml/min) | Urinary excretion rate of protein (mg/24 h) |
|---|---|---|---|---|---|
| 14 | CON | 372±9 | 34.33±5.77 | 1.34±0.30 | 18.67±5.51 |
| | SHR | 320±6.37$^{*}$ | 41.33±1.53 | 1.01±0.18 | 62.83±14.82$^{††}$ |

| | | | | | |
|---|---|---|---|---|---|
| 18 | CON | 448±6 | 39.79±4.55 | 1.21±0.10 | 19.43±4.12 |
| | SHR | 353±10.42* | 46.50±7.05 | 0.94±0.11 | 99.76±24.30†† |
| 22 | CON | 415±7 | 48.96±3.58 | 0.92±0.20 | 21.67±2.08 |
| | SHR | 318.50±5.50* | 49.33±6.97 | 0.84±0.19 | 92.77±21.59†† |
| 26 | CON | 421±10.51 | 43.55±7.78 | 1.01±0.30 | 20.88±2.74 |
| | SHR | 314.25±9.39* | 50.67±7.09 | 0.72±0.13 | 142.59±45.50†† |
| 30 | CON | 439±6 | 43.50±8.26 | 1.02±0.11 | 21.67±3.64 |
| | SHR | 314.5±27.36* | 50.33±5.68 | 0.70±0.19 | 164±32.64†† |

*$P < 0.05$, **$P < 0.01$ *vs.* the body weight of the control group, †$P < 0.05$, ††$P < 0.01$ *vs.* urinary excretion rate of protein in the control group.
